# Hesperidin Induces Paraptosis Like Cell Death in Hepatoblatoma, HepG2 Cells: Involvement of ERK1/2 MAPK

**DOI:** 10.1371/journal.pone.0101321

**Published:** 2014-06-30

**Authors:** Silvia Yumnam, Hyeon Soo Park, Mun Ki Kim, Arulkumar Nagappan, Gyeong Eun Hong, Ho Jeong Lee, Won Sup Lee, Eun Hee Kim, Jae Hyeon Cho, Sung Chul Shin, Gon Sup Kim

**Affiliations:** 1 Research Institute of Life Science, College of Veterinary Medicine (BK21 plus project), Gyeongsang National University, Gazwa, Jinju, Republic of Korea; 2 Department of Internal Medicine, Institute of Health Sciences, Gyeongsang National University School of Medicine, Gyeongnam Regional Cancer Center, Gyeongsang National University Hospital, Jinju, Republic of Korea; 3 Department of Nursing Science, International University of Korea, Jinju, Republic of Korea; 4 Department of Chemistry, Research Institute of Life Science, Gyeongsang National University, Jinju, Republic of Korea; Institut für Pathologie, Greifswald, Germany

## Abstract

Hesperidin, a natural flavonoid abundantly present in *Citrus* is known for its anti-cancer, anti-oxidant and anti-inflammatory properties. In this study we examined the effect of hesperidin on HepG2 cells. HepG2 cells treated with various concentration of hesperidin undergo a distinct type of programed cell death. Cytoplasmic vacuolization, mitochondria and endoplasmic reticulum swelling and uncondensed chromatin were observed in hesperidin treated cells. DNA electrophoresis show lack of DNA fragmentation and western blot analysis demonstrates lack of caspase activation and PARP cleavage. It was observed that hesperidin induced cell death is nonautophagic and also activate mitogen activated protein kinase ERK1/2. Taken together, the data indicate that hesperidin induces paraptosis like cell death in HepG2 cells with the activation of ERK1/2. Thus our finding suggests that hesperidin inducing paraptosis may offer an alternative tool in human liver carcinoma therapy.

## Introduction

Liver cancer is one of the most prevalent cancers and the third leading cause of cancer related deaths worldwide [6]. Hepatoblastoma is the most common primary liver tumor in children and accounts for 25–45% of the liver tumor [43]. It is of a major concern due to the poor prognosis and low rate of long term survival. And it is also highly chemoresistant to currently available chemotherapeutic agents. Preventive approaches are therefore sorely needed.

One of the most effective cancer therapy methods is induction of apoptosis using various cytotoxic agents [13]. Apoptosis is a programmed cell death (PCD) characterized by cell shrinkage, membrane blebbing, chromatin condensation, DNA fragmentation, and the formation of apoptotic bodies [12], [34]. In recent years, alternative types of PCD have also been described. Among these paraptosis which is a non-apoptotic PCD has been a new area of interest in the study of cancer related therapy. Unlike apoptosis, paraptosis is characterized by cytoplasmic vacuolation that begins with progressive swelling of mitochondria and endoplasmic reticulum. In this type of cell death, the formation of apoptotic bodies, or other characteristics of apoptotic morphology such as chromatin condensation and DNA fragmentation is absent. It typically does not response to caspase inhibitors z-VAD.fmk, BAF, p53, xiap, Bcl-XL nor does it involve activation of caspases [30], [37]. Paraptosis has also been described to be mediated by mitogen-activated protein kinases [31] and can also be triggered by the TNF receptor family member TAJ/TROY [35].

According to the WHO about 65% of the world's population relies on plant derived traditional medicines for their primary health care [5]. The use of natural products as therapeutic agents against cancer has become very popular in the recent years considering the toxicity of chemotherapeutics. Natural products are considered to be safe and also reduce the mutagenicity in normal cells [19]. In our previous studies we have also reported *Citrus aurantium* extracts, *Scutellaria baccalensis* extracts and polyphenolic extracts of *Lonicera japonica* to possess anticancer activity in human gastric cancer, lung cancer and liver cancer cells respectively [16], [26], [27].

**Figure 1:**
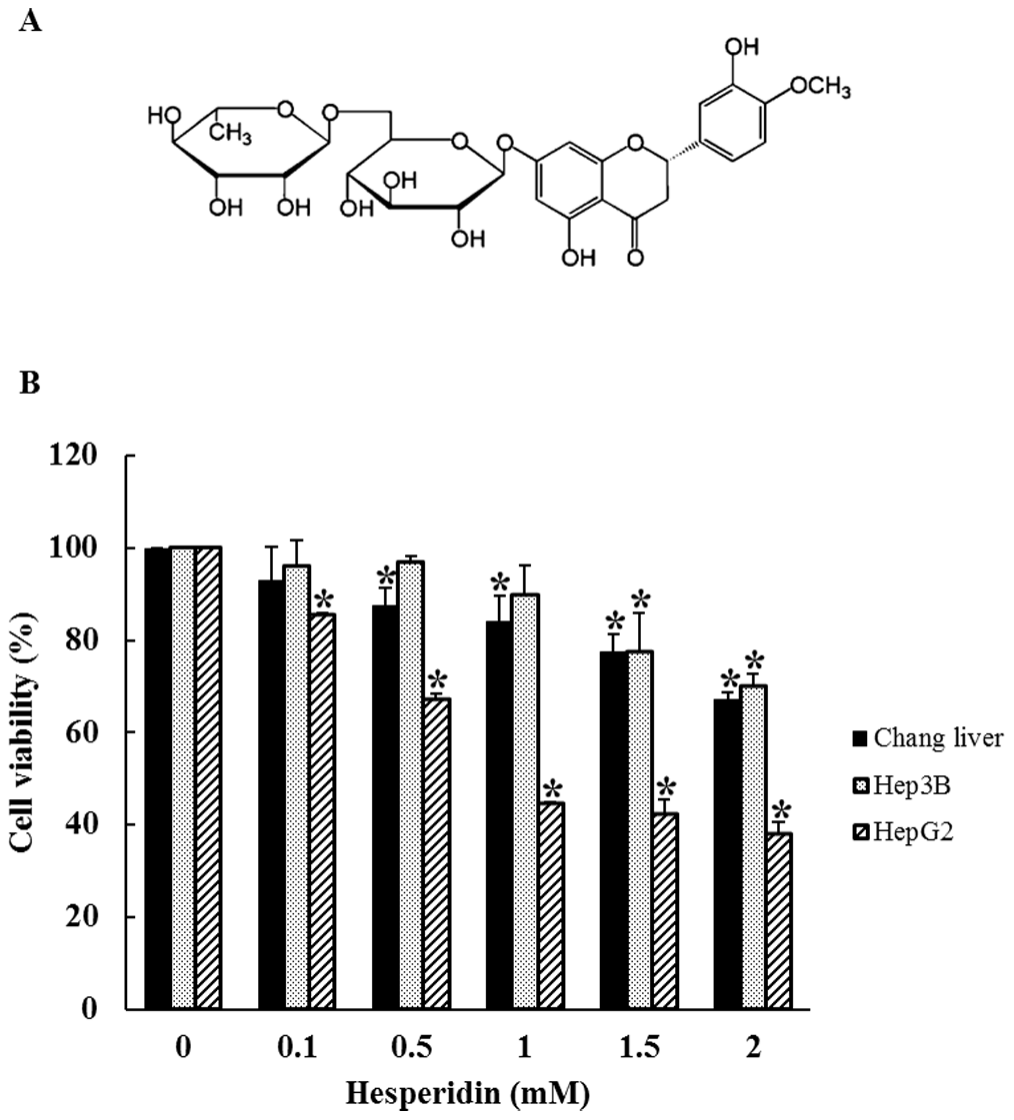
Hesperidin structure and cell viability of HepG2, Hep3B and Chang Liver cells. (A) Structure of Hesperidin. (B) Cell viability of HepG2, Hep3B and Chang Liver cells. HepG2 Hep3B and Chang Liver cells were treated with various concentration of hesperidin for 24 h and viability was determined by MTT assay. Data represent the mean ± SD of three replicates independent experiments. The asterisk (*) indicates a significant difference from the control group (*p<0.05).

Flavonoids are natural polyphenolic compounds widely occurring in fruits and vegetables. And in the recent years the use of flavonoids as anti-cancer compounds has received considerable attention [1], [22]. The flavonoid is sub-grouped into flavones, flavanols, isoflavones, flavanols, flavanones and flavanonols [28]. Hesperidin (5, 7, 3′-trihydroxy-4′-methoxy-flavanone7-rhamno glucoside) (Fig. 1A) is a flavanone glycoside widely found in *Citrus* fruits and vegetables [9]. Hesperidin has reported to exhibit diverse biological and pharmacological properties including antianalgesic, anti-inflammatory [7], antidepressant [29], antioxidant and anticarcinogenic activity [10], [38]. Hesperidin inducing apoptosis has been reported in various cancer cells including colon, pancreatic and mammary cancer cells [23], [25] but that of inducing paraptosis is still yet to be explored.

The main aim of the study was to determine the effect of hesperidin on HepG2 cells and to evaluate its anticancer potential. In the present study we observed that hesperidin induces cytoplasm vacuolation and mitochondrial swelling in HepG2 cells. In addition we also observed the non-involvement of caspase, AIF, cathepsin D, lack of apoptotic body formation, chromatin condensation and DNA fragmentation. It was also observed the cell death in HepG2 cells were nonautophagic. The results suggest that hesperidin induces paraptosis like cell death in HepG2 cells with the activation of ERK1/2 protein kinase. And as far as we know this would be the first reported study of hesperidin inducing paraptosis like cell death in HepG2 cells.

## Materials and Methods

### Chemicals and Reagents

Dulbecco's Modified Eagles medium (DMEM) media was purchased from Hyclone (Logan, UT, USA). Antibiotics (streptomycin/penicillin) and fetal bovine serum (FBS) were obtained from Gibco (Grand Island, NY, USA). 3-(4,5-Dimethylthiazol-2-yl)-2,5-diphenyltetrazolium bromide (MTT), Propidium iodide (PI), DiOC_6_ and hesperidin were purchased from Sigma–Aldrich (St. Louis, MO, USA). Goat anti-mouse and goat anti-rabbit secondary antibody were purchased from Enzo Life Sciences. 4′, 6-Diamidino-2-phenylindole (DAPI) was purchased from Vector Laboratories Inc. (Burlingame, CA). Pro-caspases 3 and active caspases 3, ERK1/2 and pERK1/2, AIF, cathepsin D, LC3B and PI3K inhibitor LY294002 were purchased from Cell Signaling (Beverly, MA, USA). The pan-caspase inhibitor, z-VAD.fmk was purchased from Promega (Madison, WI, USA) and U0126 ERK1/2 inhibitor was purchased from Tocris Biosciences (Bristol, UK)

### Cell Culture and Treatment

Liver cancer cells, HepG2, Hep3B and normal human liver cell, Chang liver cell lines were obtained from the Korean Cell Line Bank (Seoul, Korea). The cells were maintained in DMEM supplemented with 10% heat inactivated FBS and 1% penicillin/streptomycin at 37°C in a 5% CO_2_ incubator. Cells were treated with vehicle alone (1% DMSO) or 0.1, 0.5, 1, 1.5 and 2 mM of hesperidin dissolved in 1% DMSO.

### Cell Viability Assay

Cell viability was determined using MTT assay. HepG2, Hep3B and Chang liver cells were seeded at a density of 1×10^5^ cells/well in 12 well plates. After overnight incubation at 37°C in a 5% CO_2_ incubator cells were treated with 0.1, 0.5, 1, 1.5 and 2 mM of hesperidin or vehicle alone. MTT assay was carried out after 24 h of incubation. 100 µl of 0.5% (w/v) MTT dissolved in 1X PBS was added to each well and incubated for 3 h at 37°C. The medium was aspirated and the formazan contained in the cell was solubilized by 500 µl of DMSO. After 15 min of shaking, absorbance at 540 nm was measured with a microplate reader. Cell viability was expressed as a percentage of proliferation versus controls (untreated cells), which was set at 100%.

### Cell cycle analysis

Flow cytometer was used to analyze the cell cycle distribution. Cells grown overnight were treated with 0.1, 0.5, 1, 1.5 and 2 mM hesperidin or vehicle alone for 24 h in complete media. Whole HepG2 cells were harvested and fixed with 70% ethanol for 1 h at 4°C. Fixed cells were washed in phosphate-buffered saline (PBS). Cells were then incubated with 1 U/ml of RNase A (DNase free) and 5 µg/ml of propidium iodide (PI; Sigma–Aldrich) for 30 min at 4°C in the dark. FACS Calibur flow cytometer (BD Biosciences, Franklin Lakes, NJ, USA) was used to analyze the cell cycle distribution. Approximately 10000 cells were analyzed for each sample.

### DAPI staining

HepG2 cells were seeded on 12 well plates. After overnight incubation at 37°C the cells were treated with 0, 0.1 mM, 1 mM and 2 mM hesperidin. After 24 h incubation the cells were fixed with 37% formaldehyde and 95% ethanol (1∶4) for 10 min at RT. The cells were then washed with PBS and stained with DAPI. The stained cells were examined through a fluorescence microscope (Leica).

### Electron Microscopy Analysis

For the transmission electron microscopy analysis (TEM) the cells were seeded in a 100 mm dish and incubated with vehicle or 1 mM hesperidin for 24 h and 48 h. The cells were collected and fixed in 4% formaldehyde and 1% glutaraldehyde phosphate buffer (1∶1) for 32 h at 4°C. The fixative was pipetted and replaced with 8% sucrose in 1X PBS, followed by post fixation with 1% osmium tetraoxide for 1 h at 4°C. The cells were then washed with 1X PBS three times for 10 mim. After dehydration in 50–100% ethanol, the cells were embedded in Poly/Bed 812 resin (Pelco, Redding, CA, USA). The cells were polymerized overnight at 60°C. Ultrathin sections were stained with lead citrate and examined with Tecnai 12, FEI transmission electron microscope.

### DNA Fragmentation Assay

DNA was isolated using GeneAll assay kit (Seoul, Korea) according to the manufacturer's protocol. Briefly control and hesperidin treated cells were pelleted and were lysed by adding proteinase K and 200 µl of lysis buffer provided in the kit. The samples were incubated at 56°C until complete lysis. 200 µl of lysis buffer was again added and incubated at 70°C for 10 min. After adding absolute ethanol to the samples, the mixture was transferred to SV column and centrifuge at 6000 rpm to collect the DNA. The column was washed twice with washing buffer and centrifuged at full speed to dry the membrane and remove residual ethanol which may interfere with subsequent reactions. DNA was eluted in 50 µl of elution buffer supplied with the kit and collected in fresh eppendorf tube. The DNA samples were then subjected to 1.5% agarose gel electrophoresis at 100 V for 2.5 h at room temperature. Tris acetate EDTA was used as the running buffer and DNA bands were visualized under UV light.

### Mitochondrial Membrane Potential Assay

Overnight grown HepG2 cells incubated with vehicle (as control) or in presence of 1 mM hesperidin for 24 h and 48 h at 37°C in complete media were harvested and washed with 1X PBS. Washed cells were stained with DiOC_6_ (3,3′-dihexyloxacarbocyanine iodide) dye and were analyzed by flow cytometry (FACS Calibur BD Biosciences, Franklin Lakes, NJ, USA).

### Western blot analysis

Overnight grown HepG2 cells incubated with vehicle (as control) or in presence of 1 mM hesperidin for 24 h at 37°C were lysed overnight with lysis buffer (RIPA) containing protease inhibitor cocktail and EDTA. The extracts were then centrifuged at 13000 rpm for 30 min at 4°C to remove debris. The cytoplasmic fraction was separated using Nuclear and Cytoplasmic Extraction Reagents (NE-PER, Thermo, Waltham, MO, USA) according to the manufacturer's protocol. After being boiled with loading buffer, protein samples were separated by SDS–PAGE and then transferred onto polyvinylidene difluoride membrane, which was blocked with 5% non-fat milk for 1 h. Then, membranes were incubated with respective primary antibodies at 4°C for overnight. After washing five times, the membranes were incubated with respective horseradish peroxidase-conjugated secondary antibodies at 37°C for 3 h. Western blots were developed with ECL kit (GE Healthcare). Normalization was ensured by β-actin and each band was quantified using ImageJ software.

### Inhibitor assay

For the caspase inhibition assay overnight grown cells were first treated with 20 µM of pan caspase inhibitor, z-VAD.fmk for 1 h before treating with hesperidin. After 24 h incubation at 37°C MTT assay was carried out as described in section 2.3. To study the effect of MAPKs on hesperidin induced cell death in HepG2 cells 10 µM U0126 (a specific ERK1/2 inhibitor) was pretreated for 2 h prior to hesperidin treatment. Western blot and TEM were performed as described in the previous section western blot analysis and electron microscopy analysis.

### Statistical analysis

Data are expressed as the mean ± standard deviation (SD) of a minimum of three replicates independent experiments. A Student's t-test was performed using SPSS version 10.0 for Windows (SPSS, Chicago, IL, USA). The level of statistical significance was set at p<0.05.

## Results

### Hesperidin inhibited cell proliferation of HepG2 cells

To determine the effect of hesperidin on cell proliferation, HepG2, Hep3B and Chang liver cells were seeded on 12 well plates and treated with various concentration of hesperidin or vehicle alone for 24 h and MTT assay was carried out. Chang liver cell was used as a normal liver cell line. After 24 h of incubation cell viability decreases dose dependently with IC_50_ value of ∼1 mM hesperidin for HepG2 cells (Fig. 1B). However, hesperidin does not decrease the cell viability of Hep3B as well as Chang liver cell lesser than 70% indicating that effect of hesperidin on Hep3B and Chang liver cell is negligible. The results demonstrate that hesperidin does not affect normal liver cells but only HepG2 cells.

### Effect of hesperidin on cell cycle distribution

**Figure 2:**
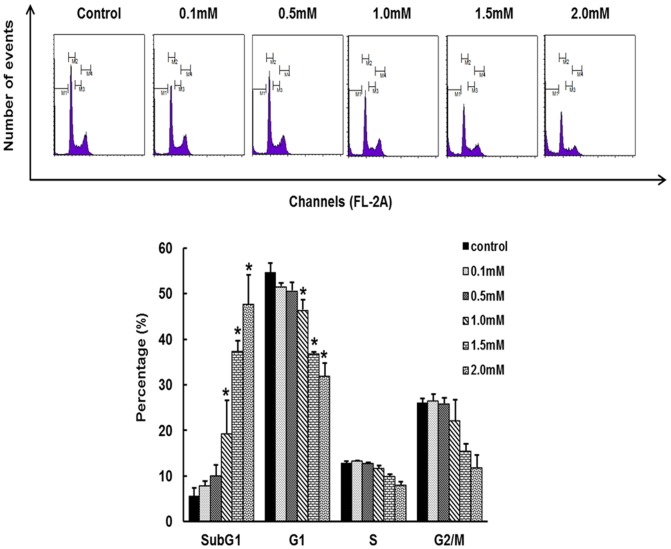
Effect of hesperidin on cell cycle distribution in HepG2 cells. Cells treated with vehicle (DMSO) or indicated concentration of hesperidin were collected and assessed by PI staining. Data represent the mean ± SD of three replicates independent experiments. The asterisk (*) indicates a significant difference from the control group (*p<0.05).

To determine the effect of hesperidin on cell cycle progression, Hepg2 cells were untreated or treated with various concentration of hesperidin for 24 h and flow cytometer analysis was performed. The subG1 phase was increased dose dependently from 5.60% in untreated control cells to 19.19% and 47.74% in cells treated with 1 mM and 2 mM hesperidin respectively (Fig. 2). Furthermore, the G1 phase cells were decreased subsequently at 1 mM, 1.5 mM and 2 mM hesperidin. The cell fraction in subG1 phase is increased with hesperidin treatment

### Hesperidin induces paraptosis like cell death in HepG2 cells

**Figure 3:**
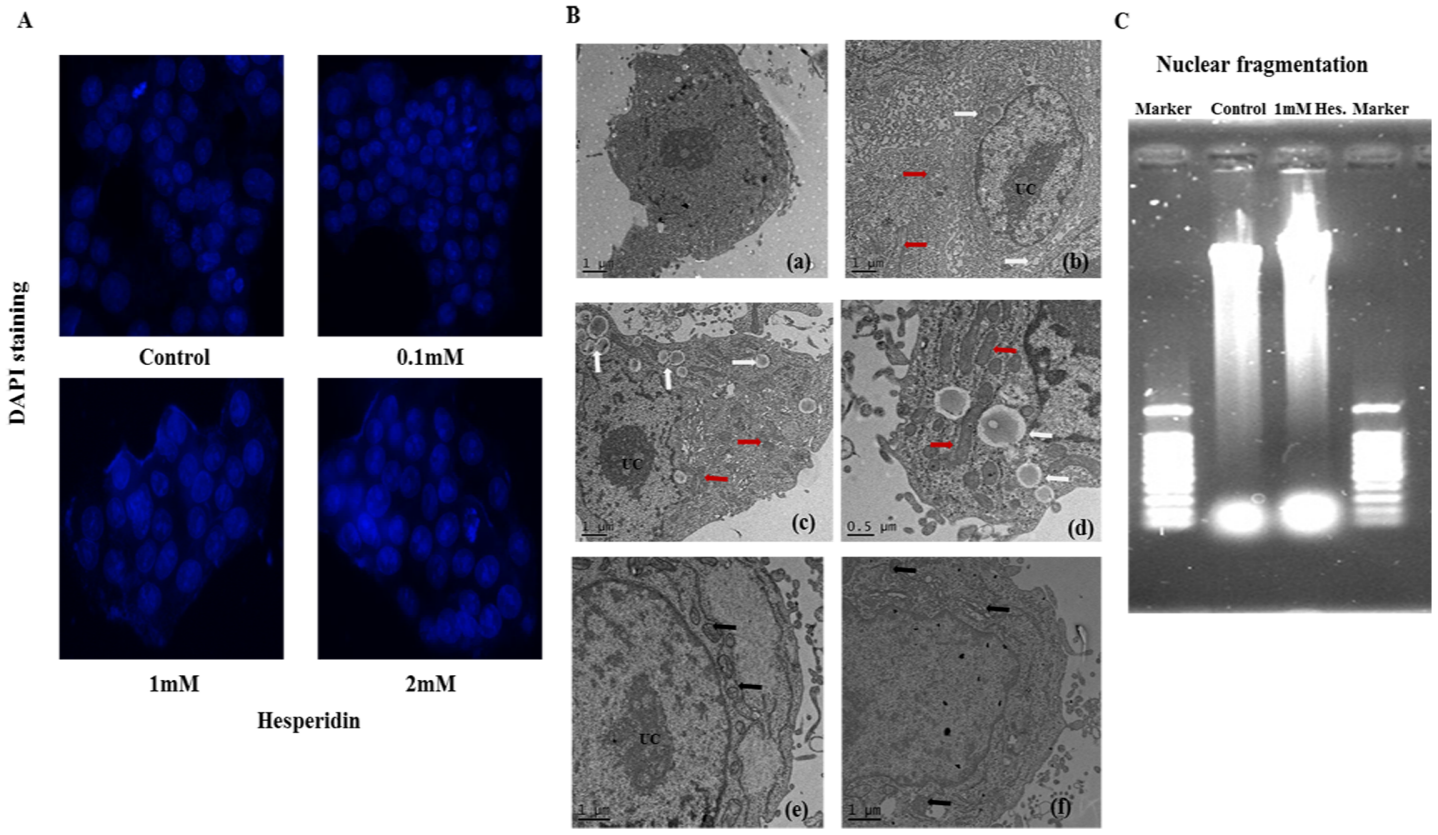
Hesperidin induces paraptosis in HepG2 cells. (A) Cells incubated with vehicle (control) or indicated concentrations of hesperidin were assessed by DAPI staining. (B) Electron microscopy showing ultrastructure of HepG2 cells untreated and treated with 1 mM hesperidin for 24 h and 48 h. (a) Control cell with normal mitochondrial and uncondensed chromatin. (b) Cells after 24 h treatment showing mitochondrial/ER swelling. (c) Cells after 48 h treatment showing cytoplasmic vacuolation and mitochondria/ER swelling. (d) Extensive swelling of mitochondrial/ER and cytoplasmic vacuolation after 48 h treatment. Inhibition of cytoplasmic vacuolation and swelling of mitochondrial/ER were observed in cells were pretreated with U0126 specific ERK1/2 inhibitor for 24 h (e) and 48 h (f). White arrows indicate cytoplasmic vacuolation, red arrows mitochondrial swelling, black arrows normal mitochondria/ER and UC condensed chromatin. (C) DNA ladder was examined in untreated and hesperidin treated HepG2 cells. There was no difference between the treated and control groups.

Most literature reported cell death induced by hesperidin was due to apoptosis [25], [17]. In order to investigate whether hesperidin induced cell death was due to apoptosis we observed the morphological changes of treated cells with DAPI and transmission electron microscope (TEM). As shown in Fig. 3A, there was no obvious change on cell nucleus accompanying with increasing concentration of hesperidin. The treated cells did not show any apoptotic characteristics or chromatin condensation. To observe the cellular ultrastructure TEM was employed. The classic apoptotic changes were absent in hesperidin treated cells, such as cell shrinkage, cytoskeletal disruption, chromatin condensation and formation of apoptotic body, which indicate that these cells did not undergo apoptosis. On the other hand extensive cytoplasmic vacuolation were observed in the treated cells. In addition, the treated cells displayed extensive swelling of mitochondria and/or endoplasmic reticulum but the nuclei were intact (Fig. 3B). These results demonstrate that hesperidin induce cell death is not apoptosis.

In the meantime, DNA fragmentation assay which is used as a biochemical analysis of apoptosis was also performed. The genome integrity was examined by agarose gel electrophoresis and there was no DNA ladder formation in the hesperidin treated cells (Fig. 3C). Taken together, these results suggest that hesperidin induced parptosis like cell death in HepG2 cells.

### Hesperidin treatment induces depletion of mitochondrial membrane potential (MMP)

**Figure 4:**
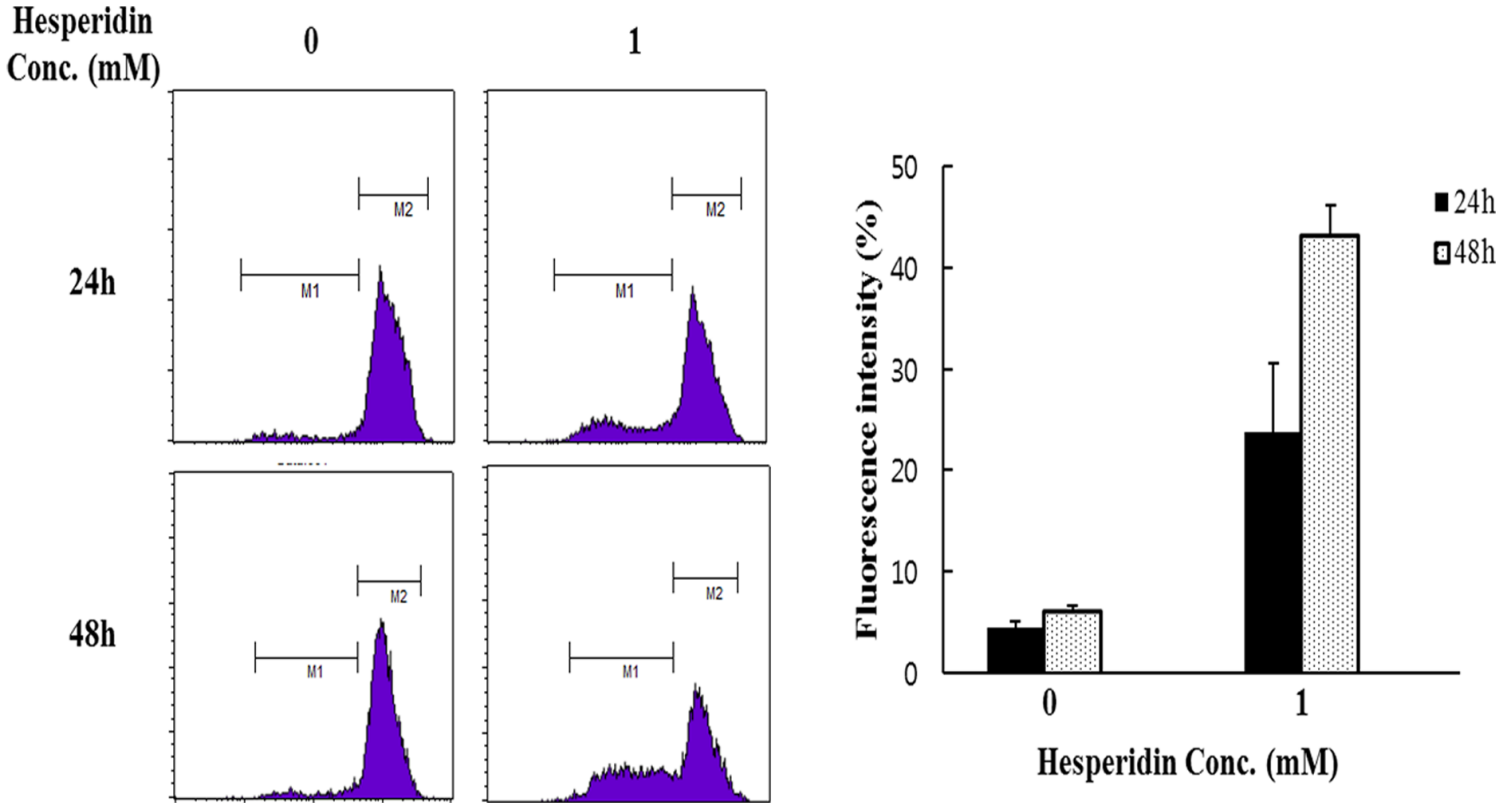
Loss of mitochondrial membrane potential induce by hesperidin. HepG2 cells untreated or treated with hesperidin for 24_6_ dye and change in fluorescent intensity was assessed by flow cytometry. Data represent the mean ± SD of three replicates independent experiments.

To study the effect of hesperidin on mitochondrial membrane potential, HepG2 cells untreated or treated with hesperidin for 24 h and 48 h were stained with DiOC_6_ dye and change of fluorescent intensity was assessed by flow cytometry. It was observed that hesperidin treatment significantly reduces the MMP of HepG2 cells (Fig. 4).

### Cell death induced by hesperidin is caspase and autophagy independent

**Figure 5:**
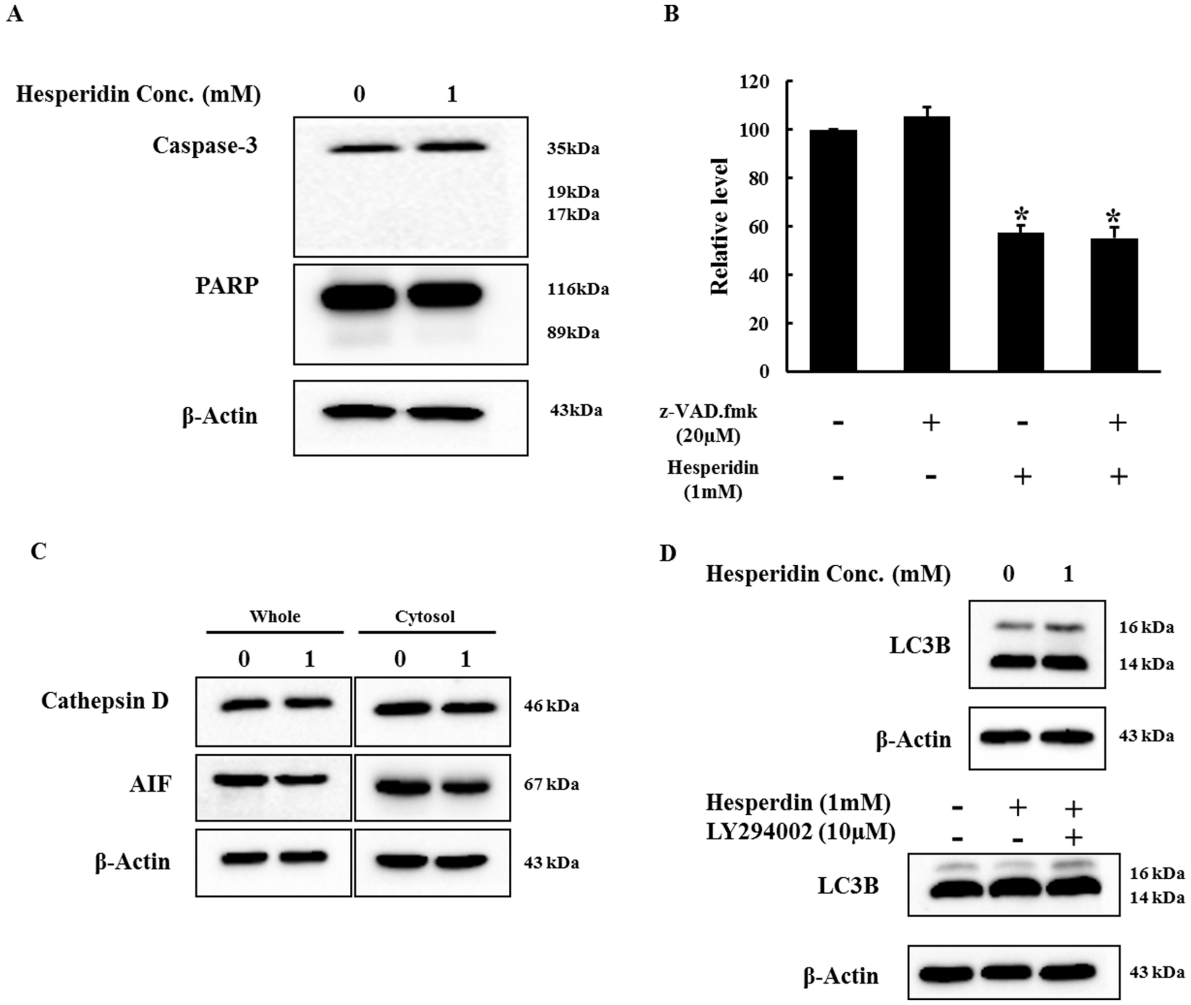
The protein expression of caspase3, PARP, cathepsin D, AIF, LC3B and cell viability with or without z-VAD.fmk. (A) Effect of hesperidin on caspase activation and PARP cleavage. Caspase 3 and PARP expression were determined by western blot. Pro caspase 3 (32 kDa) and total PARP (116 kDa) were detected but there was no active caspase 3 (17 kDa) and cleaved PARP (89 kDa) fragments. (B) Cell viability of HepG2 cells with or without z-VAD.fmk. HepG2 cells were treated with 20 µM of z-VAD.fmk for 1 h before hesperidin treatment. The cell viability was determined by MTT assay. (C) Effect of hesperidin on Cathepsin D and AIF protein expression. Cell fractionation was performed to separate the cytosolic fractions in HepG2 cells treated with 1 mM hesperidin. Cathepsin D and AIF expression were detected by western blotting in both whole cell lysate and cytosolic fractions. (D) Cell death is nonautophagic. LC3B protein expression was determined by western blot. Data represent the mean ± SD of three replicates independent experiments. The asterisk (*) indicates a significant difference from the control group (*p<0.05).

To determine whether caspase pathway is involved in hesperidin induced cell death immunoblot was carried out for hesperidin treated HepG2 cells. As shown in Fig. 5A hesperidin treated cell has lesser expression of caspases 3 precursor as compared with the control group but the active caspase were not detected. In addition, cells were per treated with z-VAD.fmk, a pan-caspase inhibitor for 1 h prior to hesperidin treatment and cell viability was checked after 24 h incubation. It was observed that the percentage of viable cell declined and hesperidin had the same effect on HepG2 cells with /without z-VAD.fmk (Fig. 5B). Moreover, there was no detection of PARP cleavage (Fig. 5A) after hesperidin treatment. In addition, Cathepsin D and AIF protein expression were also determined by western blot. It was observed that hesperidin significantly decreased the protein expression of both cathepsin D and AIF in both whole cell lysate as well as the cytoplasmic fraction (Fig. 5C). These results indicate that hesperidin induced cell death is caspase independent and does not involve the lysosome protease cathepsin D.

Furthermore, to determine whether hesperidin induced autophagy in HepG2 cells the transformation of LC3 I (cytoplasmic form) to LC3 II (preautophagosomal and autophagosomal membrane bound form) was checked. It was observed that the expression of LC3B I and LC3B II were increased in hesperidin treated cells as compared to the untreated cells but LC3B transformation (LC3 II/LC3 I ratio) was decreased in the treated cells as compared to the untreated cells (Fig. 5D). The effect of hesperidin on LC3B level was also observed in the presence of LY294002 a specific PI3K inhibitor. PI3K inhibitors are known to block LC3B processing during autophagy [11]. HepG2 cells were pretreated with 10 µM of LY294002 for 2 h prior to hesperidin treatment and the expression of LC3B was checked by western blot. As shown in Fig. 5D it was observed that LC3B transformation was not inhibited by LY294002. Taken together, these results suggest that hesperidin induced cell death in HepG2 cells is caspase as well as autophagy independent.

### Involvement of ERK1/2 MAPK in paraptosis

**Figure 6:**
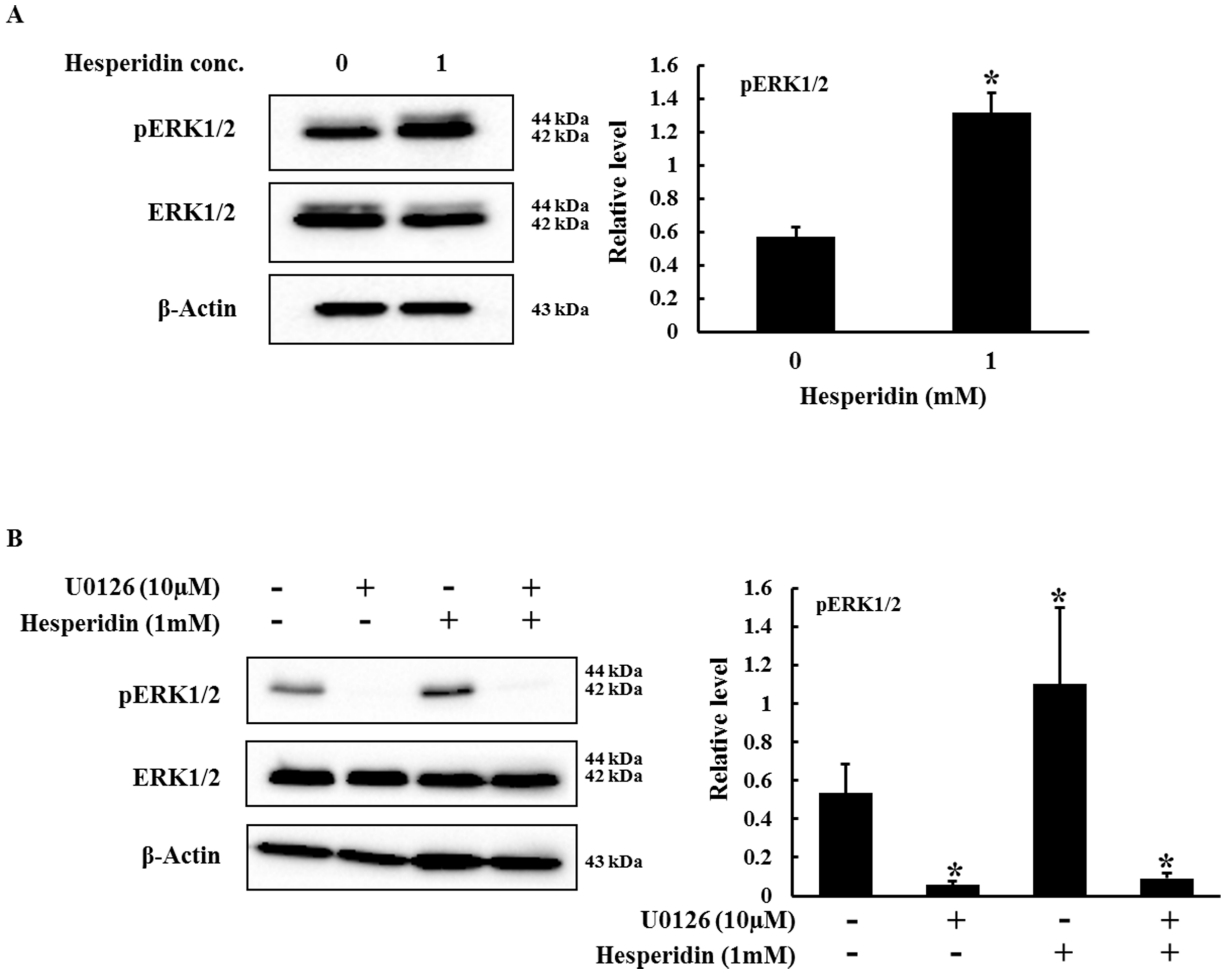
Regulation of MAPK by hesperidin. (A) HepG2 cells were untreated or treated with hesperidin for 24 h and whole cell lysate prepared from these cells were subjected to western analysis. Protein level of pERK1/2 and total ERK1/2 are shown relative to the value for untreated control cells. (B) Effect of ERK inhibitor on hesperidin induced cell death in HepG2 cells. HepG2 cells were pretreated with 10 µM of U0126 (ERK1/2 inhibitor) for 2 h before hesperidin treatment. The whole cell lysate was subjected to western analysis. Data represent the mean ± SD of three replicates independent experiments. The asterisk (*) indicates a significant difference from the control group (*p<0.05).

MAPKs have played roles in apoptosis, paraptosis as well as other form of cell death. To analyze the role of MAPKs in hesperidin induced cell death in HepG2 cells the expression of ERK1/2 was determined by western blot. Hesperidin significantly increased the protein levels of p ERK1/2 while the total ERK1/2 protein level decrease indicating that hesperidin induces paraptosis by activating ERK1/2 (Fig. 6A). In order to further analyze the role of MAPK, changes in ERK protein level was tested in the presence or absence of U0126 (specific ERK inhibitor). U0126 markedly inhibited the phosphorylation of ERK1/2 and reversed the induced effects of pERK1/2 (Fig. 6B). To further confirm the involvement of ERK1/2 in hesperidin induce paraptosis the cellular ultrastructure of HepG2 cells treated with 10 µM U0126 along with 1 mM hesperidin were observed by TEM. It was observed that U0126 inhibits the extensive cytoplasmic vacuolation as well as swelling of mitochondria and/or endoplasmic reticulum which was observed in HepG2 cells treated with hesperidin alone (Fig. 3B (e) and (f)). These results suggest that ERK1/2 is involve in hesperidin induced paraptosis in HepG2 cells.

## Discussion

Flavonoids are polyphenolic compounds and have long have been used for its anti-oxidant, anti-inflammatory and anti-tumor properties. They are richly present in *Citrus* fruits. Hesperidin is one of the most common flavonoids present in *Citrus*. It has been reported that hesperidin possess anti-cancer effects on malignant cancer cells such as colon cancer cells [25], breast cancer and prostate cancer [15] and inhibits tumor invasiveness of hepatocellular carcinoma [39], [18].

Although induction of apoptosis is the classical event for cancer suppression other types of PCD also exists in cells. The caspase independent PCD are autophagy, mitotic catastrophe, paraptosis, exitotoxicity, Wallerian degeneration and programmed necrosis [4]. Many natural anti-cancer substances such as taxol [3], [32], yessotoxin [14], ginsenoside [20], curcumin [40], honoki [36], γ-tocotrienol [42] can induce caspase independent PCD besides the caspase dependent one. The cytotoxic effect of hesperidin triggering non apoptotic mechanisms is not well understood. The present study reports for the first time that hesperidin can induce non-apoptotic programmed cell death in HepG2 cells.

In the present study we demonstrated that hesperidin induces cell death of HepG2 cells dose dependently with an IC_50_ value of ∼1 mM but it does not affect the normal cells. It was observed that hesperidin induced cell death in HepG2 cells lack typical apoptotic characteristics. Extensive cytoplasmic vacuolation, mitochondrial and endoplasmic swelling with uncondensed chromatin and lack of DNA fragmentation were observed in cells exposed to hesperidin. These results suggest that hesperidin induced cell death in HepG2 cells is paraptosis and not apoptotic. Similar results have also been described where human insulin-like growth factor I receptor (IGF-IR) induces non-apoptotic programmed cell death characterized by cytoplasmic vacuolation and resistance to apoptosis inhibitors [30]. Table 1 summarizes the observed characteristic features of HepG2 cells exposed to 1 mM of hesperidin and its comparison to that of apoptosis, necrosis and paraptosis.

**Table 1:** Characteristic morphological and biochemical traits of apoptosis, necrosis and paraptosis compared with HepG2 cells exposed to 1[14], [24], [30], [35], [42].

Parameter	Apoptosis	Necrosis	Paraptosis	HepG2 cells
Nuclear fragmentation	+	-	-	-
Chromatin condensation	+	-	-	-
Apoptotic bodies	+	-	-	-
Cytoplasmic vacuolation	-	+	+	+
Mitochondrial swelling	Sometimes	+	+	+
DNA fragmentation	+	-	-	-
Caspase 3 activation	+	-	-	-
PARP cleavage	+	-	-	-
Inhibition by z-VAD.fmk	+	-	-	-

Caspase-3 activation is considered to be a main characteristic of apoptosis but [30] reported that caspase-3 activation is absent in paraptosis. In our study we have shown that hesperidin induced cell death does not activate caspase3. In addition the pan caspase inhibitor z-VAD.fmk has no effect on hesperidin induced cell death suggesting that hesperidin induce HepG2 cell death is caspase independent. Caspase independent PCD in human colon carcinoma cells has also been reported by [42]. Similar results were also reported by [3], [24], [35].

Moreover, there was no significant poly-ADP polymerase (PARP) cleavage at 89 kDa in HepG2 cells after hesperidin treatment even though there was a decrease in the expression of intact PRAP. PARP cleavage which is considered as a hallmark in apoptosis is absent in paraptosis [30]. Lack of PARP cleavage in paraptosis induced by yessotoxin has also been reported [14]. Similar results were also observed by [24], [35].

During apoptosis cathepsin D has been reported to release from the lysosome into the cytosol which in turn releases cytochrome c thereby activating the caspase cascades [21]. And it has also been reported that cathepsin D trigger the release of AIF from the mitochondria to the cytosol [2]. In our study we have shown that cathepsin D and AIF are not involved in hesperidin induced cell death in HepG2 cells. AIF when translocate into the nucleus causes chromatin condensation and DNA fragmentation [2]. In the present study it was also observed that there is lack of chromatin condensation as well as DNA fragmentation which is supported by the non-involvement of AIF protein. Taken together, these results further confirmed that hesperidin induced cell death in HepG2 cells is non-apoptotic and caspase independent.

The present study also shows that hesperidin induced cell death is nonautophagic. LC3 processing is considered as an autophagy marker. Lack of LC3 processing and also failure of PI3K inhibitor LY294002 to inhibit LC3 processing suggests that hesperidin induced cell death in HepG2 cells is autophagy independent. Similar results were also shown by [11] where prostaglandin induces nonautophagic cytoplasmic vacuolation in colon cancer cells.

Reduction in MMP can be a checkpoint of apoptosis as it can cause the release of apoptogenic molecules from the intermembrane space into the cytoplasm [33]. In the present study we have observed significant reduction in MMP after exposure to hesperidin. But lack of caspase activation and AIF release into the cytosol was also observed suggesting that loss of MMP is not apoptosis in hesperidin induced cell death. Depletion of mitochondrial membrane potential was also observed without AIF release in calcium induced paraptotic cell death in Jurkat cells [8]. Summing up these results suggest that hesperidin induces paraptosis like cell death in HepG2 cells.

The MAPK superfamilies are serine/threonine protein kinases and play pivotal roles in a broad range of biological functions in many cell types. The ERK pathway is involved in regulating cell growth and differentiation. Apart from cell growth and apoptosis MAPK is also involved in paraptosis. Involvement of MAPK in paraptosis induced by human insulin-like growth factor I receptor (IGF-IR) has been reported by [31] while [41] reported the activation of ERK1/2 and p38 protein kinases in paraptosis like cell death induce by grape seed oligomer in human glioblastoma U87 cells. [14] also showed the phosphorylation of JNK in yessotoxin induced paraptosis in BC3H1 cells. In our study we have also shown the activation of ERK1/2 protein kinase in HepG2 cells by hesperidin. Further results show that ERK 1/2 inhibitor U0126 inhibit phosphorylation of ERK1/2, cytoplasmic vacuolation and extensive swelling of mitochondria and/or endoplasmic reticulum which suggests the involvement of ERK1/2 in hesperidin induce paraptosis in HepG2 cells.

Hesperidin is abundantly present in *Citrus* fruits. In our previous studies we have shown high content of hesperidin in *C. aurantium* [16]. In food industry *Citrus* fruits are mainly used as fresh juice and *Citrus* based juice. The waste by-products like peel, pulp, rag and seeds can be used for extracting flavonoids like hesperidin. The present study demonstrates hesperidin induces paraptosis like cell death in HepG2 cells through the phosphorylation of ERK1/2. Further studies on the molecular mechanisms by which hesperidin induces cytoplasmic vacuolation and its relation with cell death is needed. This alternative type of cell death may prove to be potential cancer therapeutics. Thus hesperidin inducing non-apoptotic cell death can be a promising chemotherapeutic agent against liver cancer.
