# Correction: Hesperidin Induces Paraptosis Like Cell Death in Hepatoblatoma, HepG2 Cells: Involvement of ERK1/2 MAPK

**DOI:** 10.1371/journal.pone.0109040

**Published:** 2014-09-23

**Authors:** The PLOS ONE Staff

There is an error in the title where Hepatoblastoma was misspelled. The correct title is: Hesperidin Induces Paraptosis Like Cell Death in Hepatoblastoma, HepG2 Cells: Involvement of ERK1/2 MAPK.

The correct citation is: Yumnam S, Park HS, Kim MK, Nagappan A, Hong GE, et al. (2014) Hesperidin Induces Paraptosis Like Cell Death in Hepatoblastoma, HepG2 Cells: Involvement of ERK1/2 MAPK. PLoS ONE 9(6): e101321. doi:10.1371/journal.pone.0101321
